# Molecular Basis of Pseudomonas syringae pv actinidiae Levansucrase Inhibition by a Multivalent Iminosugar

**DOI:** 10.1021/acs.jafc.5c01947

**Published:** 2025-05-11

**Authors:** Costanza Cicchi, Luigia Pazzagli, Paolo Paoli, Sara Campigli, Guido Marchi, Francesca Cardona, Francesca Clemente, Sara Pavone, Marta Ferraroni, Alberto Canovai, Camilla Matassini, Simone Luti

**Affiliations:** † Department of Experimental and Clinical Biomedical Sciences, 9300 University of Florence, Viale Morgagni n. 50, Florence 50134, Italy; ‡ Department of Agriculture, Food, Environment and Forestry, 9300 University of Florence, Piazzale delle Cascine n. 28, Florence 50144, Italy; § Department of Chemistry ‘Ugo Schiff’ (DICUS), 9300 University of Florence, via della Lastruccia n. 3-13, Sesto Fiorentino, Florence 50019, Italy

**Keywords:** levansucrases, polyhydroxylated pyrrolidines, DAB-1, multivalency, X-ray crystal structure, enzyme inhibition, Pseudomonas syringae pv actinidiae

## Abstract

Levansucrases are a class of polysaccharide-processing enzymes widely distributed among plant pathogenic bacteria, such as Pseudomonas syringae and Erwinia amylovora. Therefore, the modulation of levansucrase activity could represent a new strategy to reduce the microbial survival of such bacteria. Herein, we identified a tetravalent pyrrolidine iminosugar (TPIS) as the first levansucrase inhibitor described to date. TPIS reversibly inhibits sucrose hydrolysis and levan polymerization of levansucrase derived from different bacterial genotypes of P. syringae, showing competitive behavior and an inhibition constant (*K*_i_) in the micromolar range. Interestingly, the monovalent pyrrolidine iminosugar (PIS) analogue shows negligible inhibition, suggesting that multivalency plays a pivotal role in the interaction with levansucrase. To gain insight into the binding mechanism, the X-ray crystal structures of the beta levansucrase isoform from P. syringae pv actinidiae (Psa) in its native form and in complex with TPIS were solved, confirming TPIS as a competitive inhibitor of levansucrases. Only a portion of TPIS, corresponding to one chain of the tetravalent iminosugar derivative, was visible in the electron density maps. Nevertheless, our structural data provided an adequate comprehension of the inhibitor/enzyme interactions, sufficient to exclude some of the possible inhibition mechanisms justifying a multivalent effect and pave the way for the development of new, more potent inhibitors.

## Graphical abstract

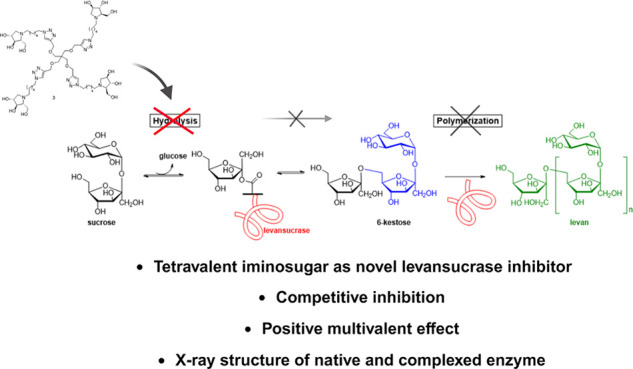

## Introduction

The glycosidase 68 family (GH68) consists of fructansucrases that play a pivotal role in sucrose use in bacteria.^1^ Among them, levansucrases (sucrose: 2,6-β-d-fructan fructosyltransferase, EC 2.4.1.10) catalyze both sucrose hydrolysis, with the release of glucose and fructose, and levan synthesis. Levan is a fructan characterized by β-2,6 glycosidic bonds with different molecular weights and degrees of β-2,1 branching.^2,3^ In fact, when sucrose is used as the substrate, levansucrases catalyze the formation of fructans with different chain lengths, including low molecular mass fructo-oligosaccharides and levan (*M*_W_ > 5 × 10^4^ Da).^4^

According to the most accepted mechanism, demonstrated for Bacillus subtilis levansucrase (SacB),^3,5^ the enzyme hydrolyzes the glycosidic bond of a sucrose molecule (which acts as a fructosyl donor) and forms a covalent fructosyl-enzyme intermediate, releasing glucose. Then, the fructosyl moiety is transferred from the enzyme to an acceptor molecule, thus resulting in the elongation of the acceptor by one fructosyl unit (Figure 1).^6^ Fructosyl acceptors can be mono-, di-, or oligosaccharides (transfructosylation reaction), and fructo-oligosaccharides, such as 6-kestose reported in Figure 1, act as initiators of the levan synthesis reactions (polymerization). When the acceptor is a water molecule, the hydrolysis of sucrose occurs, and fructose is released (hydrolysis reaction). The specificity of hydrolysis/transfructosylation reaction is strongly dependent on the reaction conditions;^5,6^ for instance, high sucrose concentrations seem to shift the reaction toward transfructosylation, probably because the disaccharide competes with water for the fructosyl-enzyme intermediate.^7^

**Figure 1:**
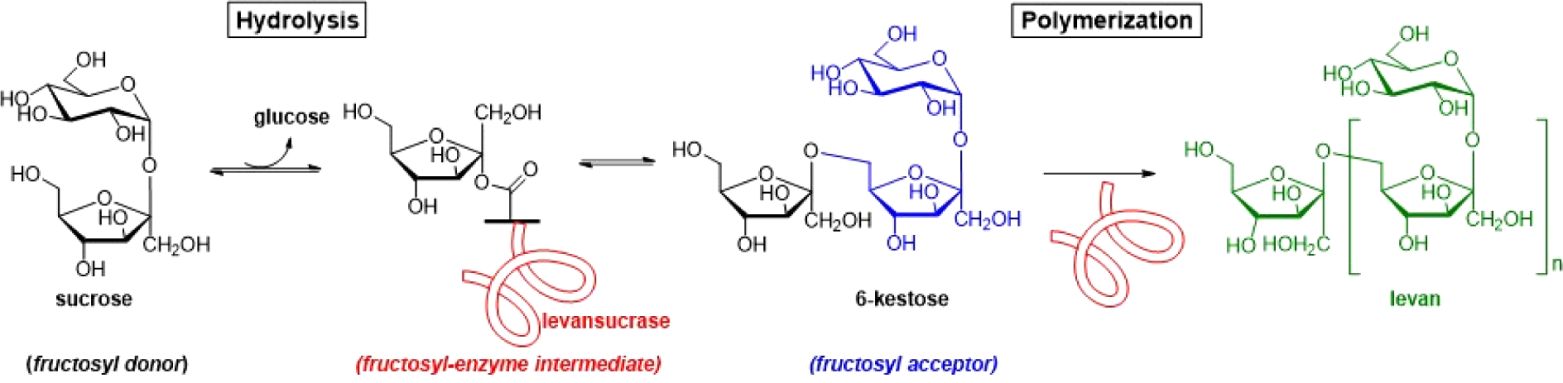
Schematic representation of the reactions catalyzed by levansucrases.

Levansucrase from Bacillus subtilis, Bacillus megaterium, Gluconacetobacter diazotrophicus, Erwinia amylovora, and Erwinia tasmaniensis was extensively purified, and their structure was described.^3,8–11^ All of them display a single domain with a conserved five-bladed β-propeller topology.^9,11,12^ Comparison of different levansucrases shows that the architecture of the central substrate-binding pocket is also conserved. In particular, the enzymatic mechanism of sucrose hydrolysis and levan synthesis involves the action of a catalytic triad including two aspartates, acting as a nucleophile and stabilizer, and a glutamate as a general acid/base catalyst.^13^

The active site surrounding loops differs depending on the enzyme’s origin: this could be responsible for differences in levan chain lengths and transfructosylation products.^4^

Genes encoding levansucrases are widely spread in bacteria and are either inducible by the presence of sucrose or constitutively expressed in bacteria that reside in sucrose-containing habitats.^14,15^ In a previous work, we focused our attention on two levansucrase isoforms (Lscβ and Lscγ) from the phytopathogenic bacterium Pseudomonas syringae pv actinidiae biovar 3 (Psa3),^16^ the major threat to kiwifruit production worldwide.^17,18^ This highly aggressive bacterium is the causative agent of the disease known as bacterial canker of kiwifruit, which causes severe losses in fruit productions every year and often leads to the death of the plants.^19^ Psa3 shares with most plant pathogens a wide range of virulence factors responsible for a successful host colonization, and one of them is the synthesis of exopolysaccharides, including levan.^20^

Interestingly, despite a sequence similarity of 95.88% (Figure S1), Lscβ and Lscγ exhibit distinct pH-dependent behavior: while Lscγ hydrolyzes sucrose at a constant rate regardless of the reaction pH, Lscβ has the highest sucrose hydrolysis activity at pH 7.0; on the contrary, Lscγ has a greater polymerization action at pH 5.0, suggesting a possible different role of the two isoforms in Psa3 physiology.^16^

Though a great effort has been made to improve bacterial levan synthesis to take advantage of its wide range of biotechnological applications,^21,22^ there is still much to unveil about the multiple physiological and pathological roles of sucrose exploitation by plant pathogens. Indeed, there is increasing evidence of the complex relationship between mono- and polysaccharides and plant disease development.^23–25^

Therefore, we chose Lscβ and Lscγ to investigate the possibility of modulating bacterial sugar metabolism and survival^26^ by inhibiting this class of enzymes. In fact, the sucrose hydrolysis inhibition of Lscβ and Lscγ can be a good model to understand the catalytic mechanism and physiological role of these enzymes. To date, the role of levan has only been hypothesized, with suggestions pointing to its involvement in biofilm formation and protection against environmental stress, or its role as a carbohydrate reserve.^14^ The development of inhibitors targeting levansucrases could provide valuable insights into the biological function of levan. We envisaged that iminosugars,^27^ i.e., natural or synthetic nitrogen-containing glycomimetics widely known to act as glycosyltransferase inhibitors,^28^ were suitable candidates to serve this role.

In this study, we identified the first levansucrase inhibitor described to date, which is a tetravalent pyrrolidine iminosugar derivative. In order to explore the spectrum of activity of the identified compound, we tested its inhibitory activity on Psa3 (strain KL103) lysates and, for comparative purposes, on those of P. syringae pv actinidiae biovar 5 (CFBP8414) and P. syringae pv phaseolicola (CFBP1390). The inhibitor kinetic parameters (IC_50_ and *K*_i_) for the two reactions (sucrose hydrolysis and levan synthesis) and against both isoforms were determined. Moreover, the X-ray crystal structures of both Lscβ alone and in complex with the inhibitor were solved.

## Materials and Methods

### Synthesis of Compounds **1**–**4**

The pyrrolidine iminosugars **1**–**4** (Figure 2) were synthesized starting from commercially available carbohydrate d-arabinose (purchased from Biosynth Carbosynth, UK). In particular, pyrrolidine DAB-1 iminosugar (**1**),^29^ the pseudodisaccharide **2**,^30^ the tetravalent pyrrolidine iminosugar TPIS (**3**),^31^ and its monovalent reference compound, namely the monovalent pyrrolidine iminosugar PIS (**4**),^32^ were synthesized according to procedures previously reported by some of us.

**Figure 2:**
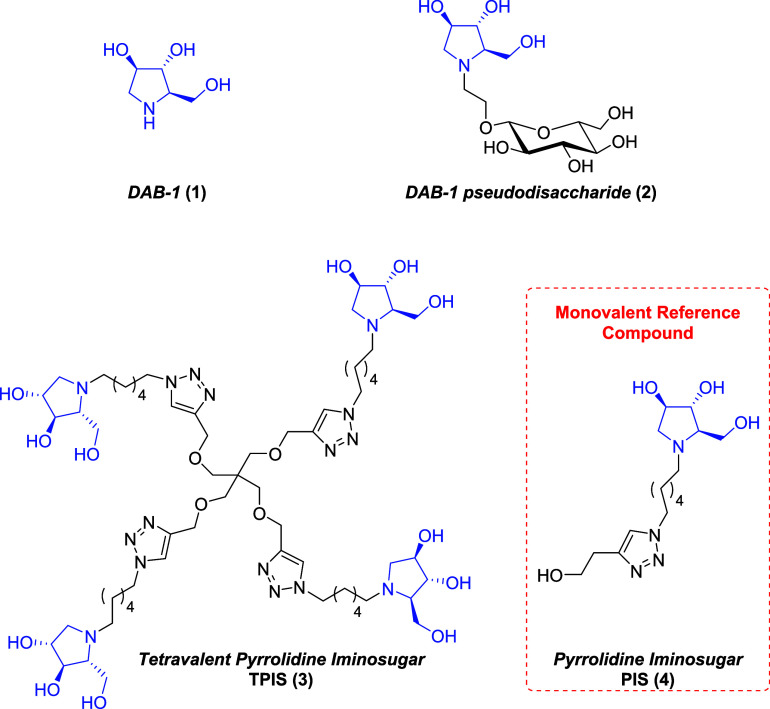
Pyrrolidine iminosugars **1**–**4** tested on Psa3 levansucrase Lscβ and Lscγ.

### Production and Purification of Lscβ and Lscγ

Lscβ and Lscγ were produced and purified as reported by Luti et al. 2021.^16^ Briefly, the *lscβ* and *lscγ* genes of Psa3 KL103 were cloned in the pNIC28-Bsa4 plasmid and used to transform E. coli BL21 (DE3) cells (New England Biolabs). At *A*_600_ (absorbance at 600 nm) = 0.6, protein expression was induced by 0.4 mM IPTG for 20 h at 18 °C. Then, cells were harvested by sonication, and recombinant proteins were purified using IMAC Sepharose High Performance (Ge Healthcare) charged with Ni^2+^. Subsequently, the proteins were subjected to TEV (Tobacco Etch Virus protease) treatment to remove the 6His-tag and further purified by gel chromatography on a HiLoad 16/600 Superdex 200 pg column driven by an Akta Pure 25 L system (GE Healthcare, Waukesha, WI, USA). Pure proteins were stored in 10 mM Tris/HCl at pH 7.0 at −80 °C until further analysis.

### Sucrose Hydrolysis Assay

The enzymatic assays to detect the inhibitory potential of the compounds on sucrose hydrolysis activity of Lscβ and Lscγ were performed according to Luti et al. 2021 ^16^ in a total volume of 100 μL, using a Bis/Tris buffer (50 mM, pH 7.0). The experiments were performed at an enzyme concentration of 0.03 μg/μL with 60 mM sucrose and a final inhibitor concentration (compounds **1**–**4**) of 100 μM, on a 10-min time frame. After the reactions were stopped, glucose concentration was measured using the DNS (3,5-dinitrosalicylic acid) method to determine spectrophotometrically the inhibitory activity of each compound.^33^ The enzymatic activity, measured as μmol/min·mL, was expressed as $\%\;\mathrm{residual enzymatic activity}=\left[\frac{A_{i}}{A_{c}}\right]\times 100$ , where *A*_i_ is the absorbance of the samples with the inhibitors and *A*_c_ is the absorbance of the control.

### Determination of IC_50_ Values of Compounds **1**–**4**

The inhibition levels of compounds **1**–**4** were analyzed by measuring their IC_50_. To quantify the IC_50_ values, we set up different enzymatic reactions at various concentrations of the inhibitor, and we monitored the effects of such concentrations on the enzymatic activity. The assay system was prepared as described above using a Bis/Tris buffer at pH 7.0 and 60 mM sucrose as the substrate. The inhibitor concentrations tested for both enzymes were 0, 0.01, 0.1, 0.5, 1, 10, 25, 50, 100, 200, 400, and 800 μM. Glucose released by the two enzymes was quantified using the DNS method and a microplate spectrophotometer. IC_50_ was defined as the concentration of a compound causing 50% inhibition, and it was calculated by drawing a dose–response graph for each compound using GraphPad Prism version 8.0 for Windows, GraphPad Software, San Diego, California, USA (www.graphpad.com). The response was expressed on the Y-axis as a percentage of enzymatic activity, and the substrate concentration was set on the X-axis as log­[sucrose].

### Mechanism of Inhibition of TPIS (**3**) on Lscβ and Lscγ Sucrose Hydrolysis

The inhibitory mechanism of TPIS (**3**) was determined by studying the dependence between the main kinetic parameters (*K*_Mapp_ and *V*_maxapp_) and the inhibitor concentrations. Data obtained were fitted using the Michaelis–Menten equation and a nonlinear fitting software. Then, data were analyzed using the double reciprocal plot (Lineweaver–Burk method). Secondary plots were used to calculate the *K*_i_ values by using GraphPad Prism version 8.0. In detail, the inhibitory mechanism of TPIS (**3**) was analyzed using 0, 25, 60, and 100 μM inhibitors for Lscβ and 0, 10, 25, and 100 μM for Lscγ. For each concentration, we set the sucrose concentration to 0, 10, 30, 50, 100, 200, 400, and 600 mM, and reactions were performed as previously described using an enzyme concentration of 0.02 μg/μL. After the reactions were stopped, glucose from enzymatic hydrolysis was quantified using the DNS method.

### Crystallization and Data Collection

Lscβ was crystallized at 296 K using the hanging drop vapor diffusion method (VDX 24-well crystallization plates). The concentration of the protein was 10 mg/mL in 10 mM Tris-HCl pH 7.0. Initial crystallization conditions were found using SG1 Screen (Molecular Dimensions), and then, they were optimized. The best crystals grew from a solution containing 20% PEG 3350 and 100 mM sodium thiocianate in 1 week. The crystals belong to the monoclinic space group *P*2_1_ with unit cell dimensions reported in Table S1. The asymmetric unit contains one dimer (*V*_M_ = 2.32 Å^3^/Da, solvent content 46.9%). Preparation of the enzyme–inhibitor complex was performed using another crystal form that was obtained using the SG1 Screen (Molecular Dimensions). The optimized crystallization condition contained 21% PEG 8000, 0.25 M sodium acetate and 0.1 M MES pH 6.0. These crystals belong to the monoclinic space group *C*_2_, with unit cell dimensions reported in Table S1. In this case, the asymmetric unit contains one enzyme monomer (*V*_M_= 2.63 Å^3^/Da, solvent content 53.2%). The enzyme complex with TPIS (**3**) was prepared by soaking the Lscβ crystals in the crystallization solution, where the inhibitor was dissolved at a concentration of 8 mM for 24 h. The data collections were performed at 100 K at the XRD2 beamline (Elettra Synchrotron, Trieste, Italy) using a 1.0000 Å wavelength and a Dectris Pilatus 6 M detector. Crystals were cryoprotected using the crystallization solution supplemented with 20% glycerol. Data were integrated and scaled using the program XDS.^34^ The statistics of the data collections are reported in Table S1.

### Structure Solution and Refinement

The structure of Lscβ was solved by the molecular replacement technique using the program Molrep^35^ from the CCP4 program package^36^ and the coordinates of the levansucrase from E. tasmaniensis (PDB ID: 7OSO) as a starting model, which is the closest homologue to Lscβ. The two enzymes have a sequence identity of 78.3%. The structure of the complex was solved in the C-centered monoclinic space group using the program Molrep and the native Lscβ structure in the other crystal form as a model. The *F*_o_–*F*_c_ maps, calculated with data collected on the crystal soaked in the inhibitor solution, showed electron densities that could unambiguously be attributed to the inhibitor TPIS (**3**). Coordinates of TPIS (**3**) were obtained using the program JLigand^37^ and introduced in the model. Model refinements were performed using Refmac5 ^38^ from the CCP4 package.^36^ Water molecules were added using ARP/wARP.^39^ Models were built using the program Coot.^40^ Inhibitor–enzyme interactions were analyzed using the PLIP web tool.^41^ Figures were prepared using the program Chimera.^42^ Coordinates of the protein were deposited in the PDB with codes 8QJ5 and 8QKW. The final models of native (apo) Lscβ and of the complex with TPIS (**3**) include, for each monomer, residues 20–431. The first 19 residues were not modeled due to the scarce electron density corresponding to this part of the enzyme.

### Dynamic Light Scattering

Size distribution analysis was performed at 25 °C with a Malvern Zetasizer Nano S Dynamic Light Scattering (DLS) device (Malvern Panalytical, Malvern, United Kingdom) using protein (Lscβ) at a concentration of 0.1 μg/μL in 50 mM Bis/Tris pH 7.0 buffer with or without TPIS (**3**). The protein solution was centrifuged at 13,000 × *g* for 15 min at 4 °C, filtered with Whatman Anotop 0.02 μm cutoff filters (Millipore Sigma), and analyzed considering the refractive index and viscosity of the dispersant. At the ending of the protein alone spectrum acquisition, TPIS (**3**) at a final concentration of 100 μM was added to the solution, and after 10 min, the spectrum was recorded again. A 10 mm reduced-volume plastic cell was used.

### Inhibition of Levan Synthesis by TPIS (**3**)

Inhibition of levan synthesis was monitored using a turbidity assay on a microtiter plate as reported by Luti et al. 2021 ^16^ with slight modifications. The reaction was performed at 37 °C in 200 μL of 50 mM sodium acetate buffer at pH 5.0 containing 60 mM sucrose and 10 μg of purified Lscγ protein per mL of reaction mixture. The turbidity of the samples at 400 nm was recorded every 5 min using a BioTek Synergy H1 Hybrid Multimode Reader. The percent inhibition was determined at 60 min of incubation, at the maximum absorbance, and was determined as defined in the previous section, Sucrose Hydrolysis Assay. For the IC_50_ determinations, we used a 0–200 μM TPIS (**3**) concentration and defined the activity as previously reported. For macroscopic evaluation of levan synthesis inhibition, a 10 μL drop of 1 μg/μL Lscγ was applied on an agar sucrose (5% w/v) plate alone or with 100 μM TPIS (**3**). A picture was taken after 24 h at 4 °C.

### Evaluation of the Effect of TPIS (**3**) on Different P. Syringae Lysates

The strains CFBP8414 (P. syringae pv actinidiae biovar 5) and CFBP1390 (P. syringae pv phaseolicola) were purchased from the CIRM-CFBP French Collection for Plant Associated Bacteria, while KL103 (P. syringae pv actinidiae biovar 3) was isolated in Tuscany in 2015, as reported by Campigli et al., 2023.^43^

Bacterial cells^16^ were grown in 50 mL of Nutrient Sucrose (5% w/v) for 48 h at 180 rpm at 27 °C. Cell suspensions were diluted to an *A*_600_ of 1 (10^7^ cfu/mL) in water, and 1 mL aliquots were centrifuged for 5 min at 15,000 × g at 4 °C. The supernatant was discarded, while the pellet was stored at −20 °C for further analysis.^43^

To extract total protein, cell pellets were resuspended in 1 mL of ice-cold RIPA Buffer with Protease Inhibitors Cocktail, and cells were lysed by sonication using a Fisher Scientific Model 120 Sonic Dismembrator. The sonication step was carried out on ice with three pulses of 15 s at 30% amplitude, each followed by 30 s cooldown. Sonicated samples were cleared by centrifugation for 15 min at 15,000 × *g* at 4 °C. The pellet was discarded, while the supernatant was collected and stored at −20 °C for further analysis. The hydrolysis enzymatic reaction was carried out as previously described with 60 mM sucrose and 7 μL of cell lysate for 1.5 h. After the samples were boiled, glucose was quantified using the DNS method. For macroscopic evaluation of levan synthesis inhibition, a 10 μL drop of lysates was applied on an agar sucrose (5% w/v) plate alone or with 100 μM TPIS (**3**). A picture was taken after 72 h at 4 °C.

### Statistical Analysis of Sucrose Hydrolysis and Levan Polymerization Assays

The sucrose hydrolysis and levan polymerization assays to evaluate the inhibitory effect of TPIS (**3**) are presented as the mean ± standard deviation. Statistical analysis was performed using one-way ANOVA or *t*-test, as specified in the text. Statistical significance was set at *p* < 0.05.

## Results and Discussion

### Evaluation of the Inhibitory Activity of Compounds **1**–**4** on Recombinant Levansucrases

Iminosugars are natural or synthetic carbohydrate analogues in which the endocyclic oxygen is replaced by a nitrogen atom, and they have been widely investigated both as glycosidase and glycosyltransferase inhibitors.^27,28^ Therefore, they represent valuable candidates as levansucrase inhibitors. Starting from a collection of iminosugars with different structures readily available in our laboratories, we selected the natural iminosugar 1,4-dideoxy-1,4-imino-d-arabinitol (DAB-1 (**1**), Figure 2) as a key feature, which was synthesized according to a reported procedure.^29^ Indeed, the inhibitory activity of DAB-1 (**1**) toward glycosidases and glycosyltransferases^28,44–46^ has been previously demonstrated, and the characteristic “*all trans*” configuration of its hydroxyl groups could in principle mimic both the glucose and the fructose unit of sucrose. Likewise, the pseudodisaccharide **2** ^30^ (Figure 2), which bears a glucose unit linked to DAB-1 through an ethyl spacer and was reported as a potent insect trehalase inhibitor, has been chosen.^30^ Finally, due to the recently emerged propension of some glycosidases to accept multivalent iminosugars ligands^31,32,47,48^ the tetravalent pyrrolidine iminosugar TPIS (**3**)^31^ was tested together with its monovalent reference compound, the pyrrolidine iminosugar PIS (**4**)^32^ (Figure 2). The effectiveness of TPIS (**3**) in the inhibition of carbohydrate-related enzymes was already demonstrated toward Jack bean α-mannosidase, where it showed an IC_50_ value of 34 μM and a rp/*n* (namely the relative potency with respect to its monovalent counterpart per active unit) value of 10.^31^ The synthesis of compounds **1–4** was carried out according to the literature (see the Materials and Methods section), and their characterization was reported in Figures S2–S9.

Both hydrolysis, with glucose release, and levan polymerization might have a physiological role in bacterial development.^4,16^ We first decided to screen the activity of compounds **1**–**4** as inhibitors of the initial catalytic step of the process (hydrolysis, Figure 1), which consequently blocks the polymerization reaction. According to previous results, sucrose hydrolysis was tested at pH 7.0, at which Lscβ exhibits 2-fold higher hydrolysis activity than Lscγ.^16^

A preliminary screening performed at a 100 μM concentration of iminosugar shows that DAB-1 (**1**) and its pseudodisaccharide (**2**) do not affect Lscβ activity at all (Figure 3A). Conversely, TPIS (**3**) inhibits sucrose hydrolysis on both levansucrase isoforms, Lscβ (49 ± 5% of residual activity) and Lscγ (36 ± 2% of residual activity) at the same concentration (Figure 3A,B, respectively). Interestingly, its monovalent counterpart PIS (**4**), prepared to mimic a single branch of TPIS (**3**), does not inhibit sucrose hydrolysis on the Lscγ isoform (Figure 3B) and shows only negligible inhibitory activity (about 7%) on the Lscβ isoform (Figure 3A), which is further confirmed by the fact that increasing the PIS (**4**) concentration up to 1 mM still yields 40% residual activity (data not shown). These data suggest that the triazole-containing pendant introduced on the endocyclic nitrogen of the pyrrolidine iminosugar in PIS (**4**) is not sufficient to confer the inhibitory activity, while the presence of four iminosugar units as in TPIS (**3**) is essential for this purpose. Of note, similar results were obtained with other carbohydrate-processing enzymes such as Jack bean α-mannosidase^49^ and *N*-acetylgalactosamine-6-sulfatase,^32,50,51^ which showed a similar sensitivity to multivalent inhibitors.

**Figure 3:**
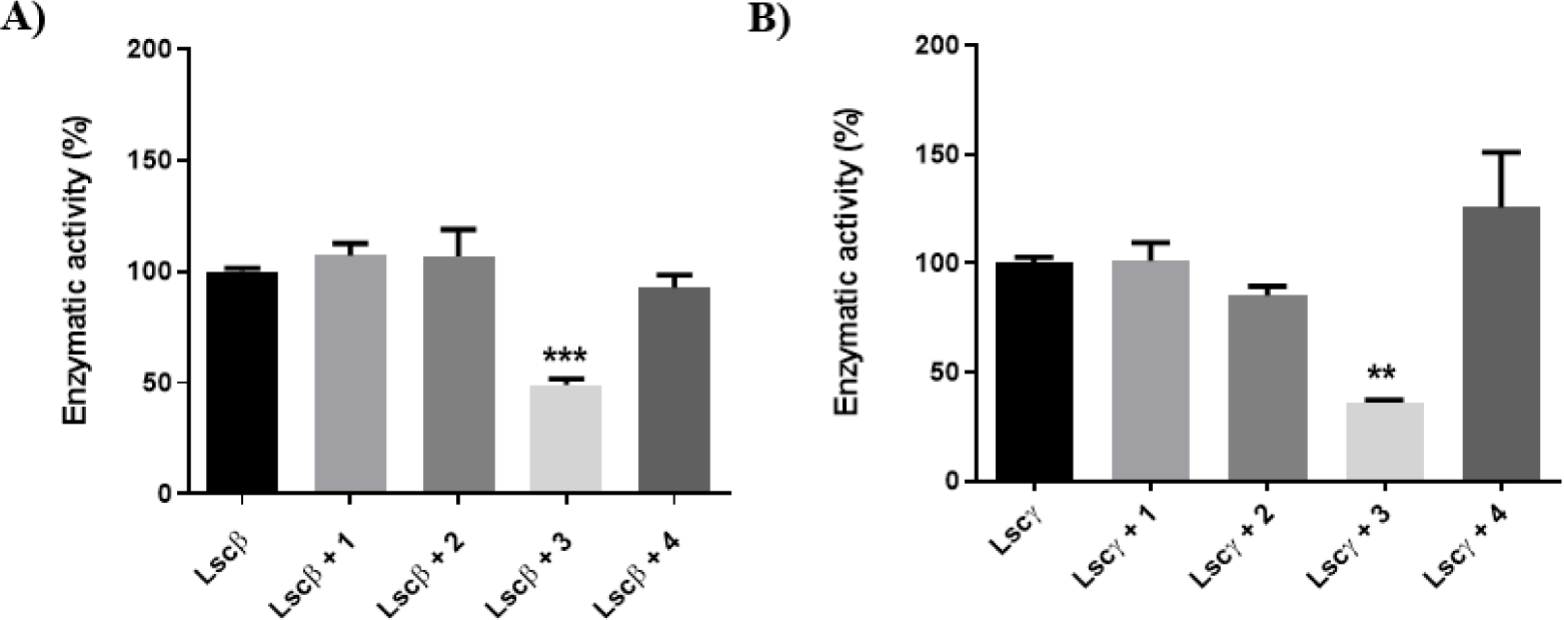
Screening of compounds **1**–**4** as potential levansucrase inhibitors. Residual enzymatic activity of Lscβ (A) and Lscγ (B) at 100 μM iminosugar, pH 7.0. Bars represent the mean of 4 replicates ± standard deviation. One-way ANOVA (** *p* ≤ 0.01; *** *p* ≤ 0.001).

### Determination of Inhibitory Parameters and Evaluation of the Inhibitory Mechanism of TPIS (**3**) on Sucrose Hydrolysis of Lscβ

To obtain further information on the inhibitory mechanism of the best inhibitor emerged in this study, namely TPIS (**3**), we studied how the main kinetic parameters *K*_Mapp_ and *V*_maxapp_ vary as a function of the inhibitor concentration. Analysis of the dose-dependent loss of activity of Lscβ at increasing concentrations of TPIS (**3**) (Figure 4A) indicates that TPIS (**3**) inactivates the sucrose hydrolysis of Lscβ showing an IC_50_ of 22 μM. In an attempt to evaluate the inhibition mechanism of TPIS (**3**), the experimental data obtained from kinetic analyses were fitted using both the Michaelis–Menten (Figure 4B) and Lineweaver–Burk equations (Figure 4C). We observed that the *K*_Mapp_ value increases, while the *V*_maxapp_ does not vary following the increase in the inhibitor concentration. These results suggest that TPIS (**3**) behaves as a competitive inhibitor of Lscβ (Figure 4D,E). Moreover, a *K*_i_ value of 29 μM was calculated, reflecting an efficient interaction between the inhibitor and the enzyme (Figure 4F). Interestingly, since the monovalent reference compound PIS (**4**) does not show any inhibitory potential on levansucrase activity, we assumed a multivalent effect of the inhibitor. Hence, the relative potency (rp) was calculated by dividing the IC_50_ of PIS (**4**) (which is higher than 1000 μM, Table 1) by the IC_50_ of TPIS (**3**) (22 μM). Further dividing the rp (45) by the valency of TPIS (**3**) resulted in an rp/*n* >11, which unequivocally demonstrates the existence of a positive multivalent effect. Such higher effectiveness of the multivalent inhibitor suggests a multimeric nature of the enzyme, as we previously reported for both Lscβ and Lscγ.^16^

**Figure 4:**
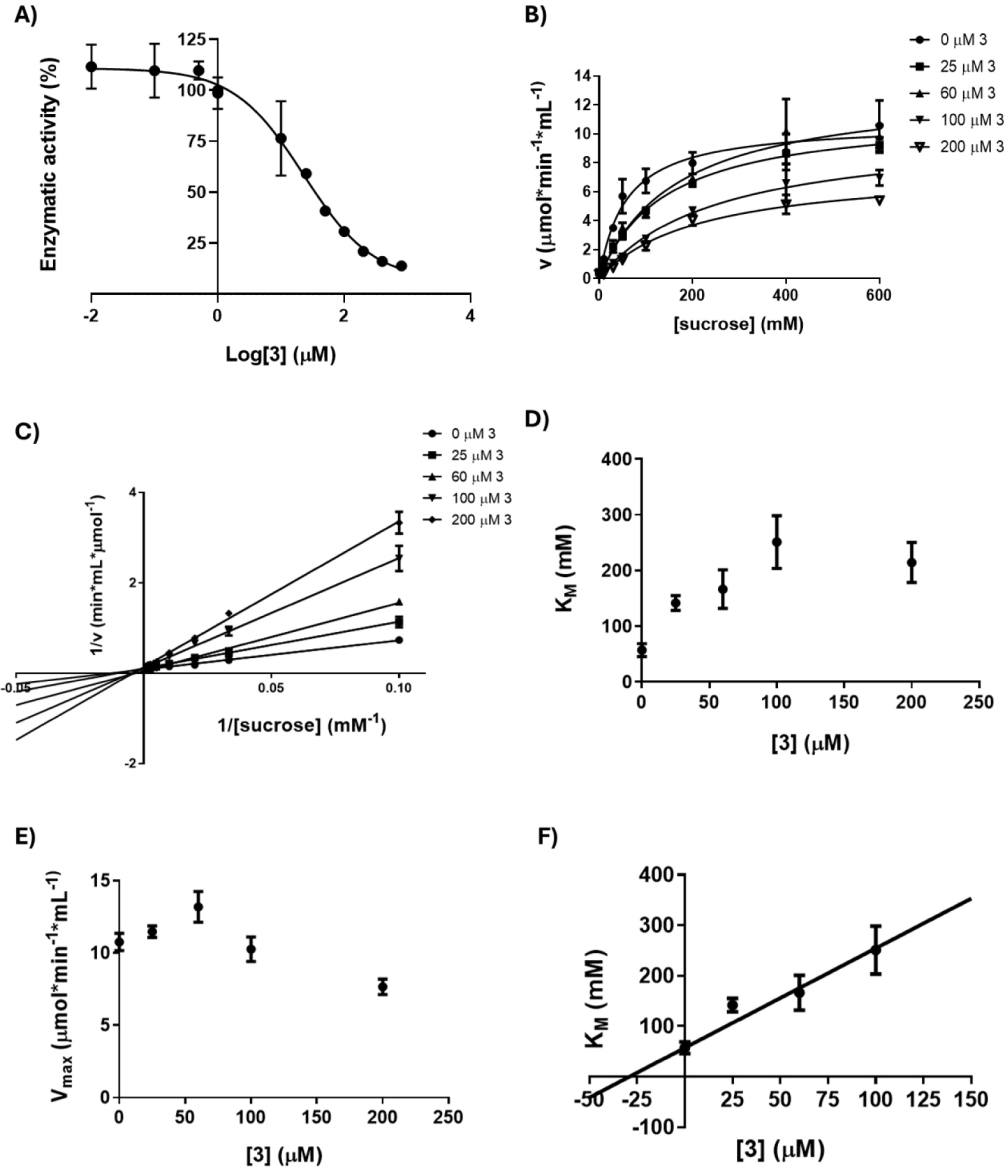
A) IC_50_ of TPIS (**3**) on sucrose hydrolysis of Lscβ. B) Michaelis–Menten plot at four different concentrations of TPIS (**3**) for Lscβ (0, 25, 60, 100 and 200 μM). C) Lineweaver–Burk plot for the detection of kinetic parameters *K*_Mapp_ and *V*_maxapp_ for Lscβ. D) *K*_Mapp_ values of Lscβ at three different concentrations of TPIS (**3**). E) *V*_maxapp_ values of Lscβ at three different concentrations of TPIS (**3**). F) Secondary plot for *K*_i_ determination. Data represent the mean of two different experiments performed in duplicate.

**Table 1:** % Residual Enzymatic Activity at 100 Μm in the Presence of Compounds **1**–**4**, IC_50_ of Compounds **1**–**4**, and *K*_i_ (Parentheses) Calculated for TPIS (**3**)

	Lscβ		Lscγ	
Compound	% Residual activity	IC_50_ (μM)	% Residual activity	IC_50_ (μM)
1	103 ± 6	>100	101 ± 12	>100
2	107 ± 24	>100	85 ± 8	>100
3	49 ± 5	22.4 (*K*_i_ 29 μM)	36 ± 5	60.9 (*K*_i_ 20 μM)
4	93 ± 12	>1000	126 ± 50	>1000
Sucrose	*K*_Mapp_ (mM) 57 ± 11		*K*_Mapp_(mM) 59 ± 12	

### Determination of Inhibitory Parameters and Evaluation of the Inhibitory Mechanism of TPIS (**3**) on Sucrose Hydrolysis of Lscγ

To investigate the inhibitory potential of TPIS (**3**) on the other levansucrase isoform, we determined both the IC_50_ value and the *K*_i_ value on Lscγ at pH 7.0. Figure 5A shows the dose-dependent loss of activity of Lscγ at increasing concentrations of TPIS (**3**). For this isoform, an IC_50_ of 60.9 μM was calculated, which was slightly higher than the IC_50_ of isoform β (Table 1). The Michaelis–Menten and Lineweaver–Burk plots (Figure 5B,C, respectively) show that the presence of TPIS (**3**) in the assay solution leads to an increase of the *K*_Mapp_ while not affecting the *V*_maxapp_ value (Figure 5D,E). This finding suggests that TPIS (**3**) behaves also as a competitive inhibitor of Lscγ versus sucrose. For this levansucrase isoform, a *K*_i_ value of 20 μM was detected (Figure 5F and Table 1). Data obtained for Lscβ and Lscγ altogether demonstrated a similar inhibitory strength and mechanism of TPIS (**3**) for both levansucrase isoforms.

**Figure 5:**
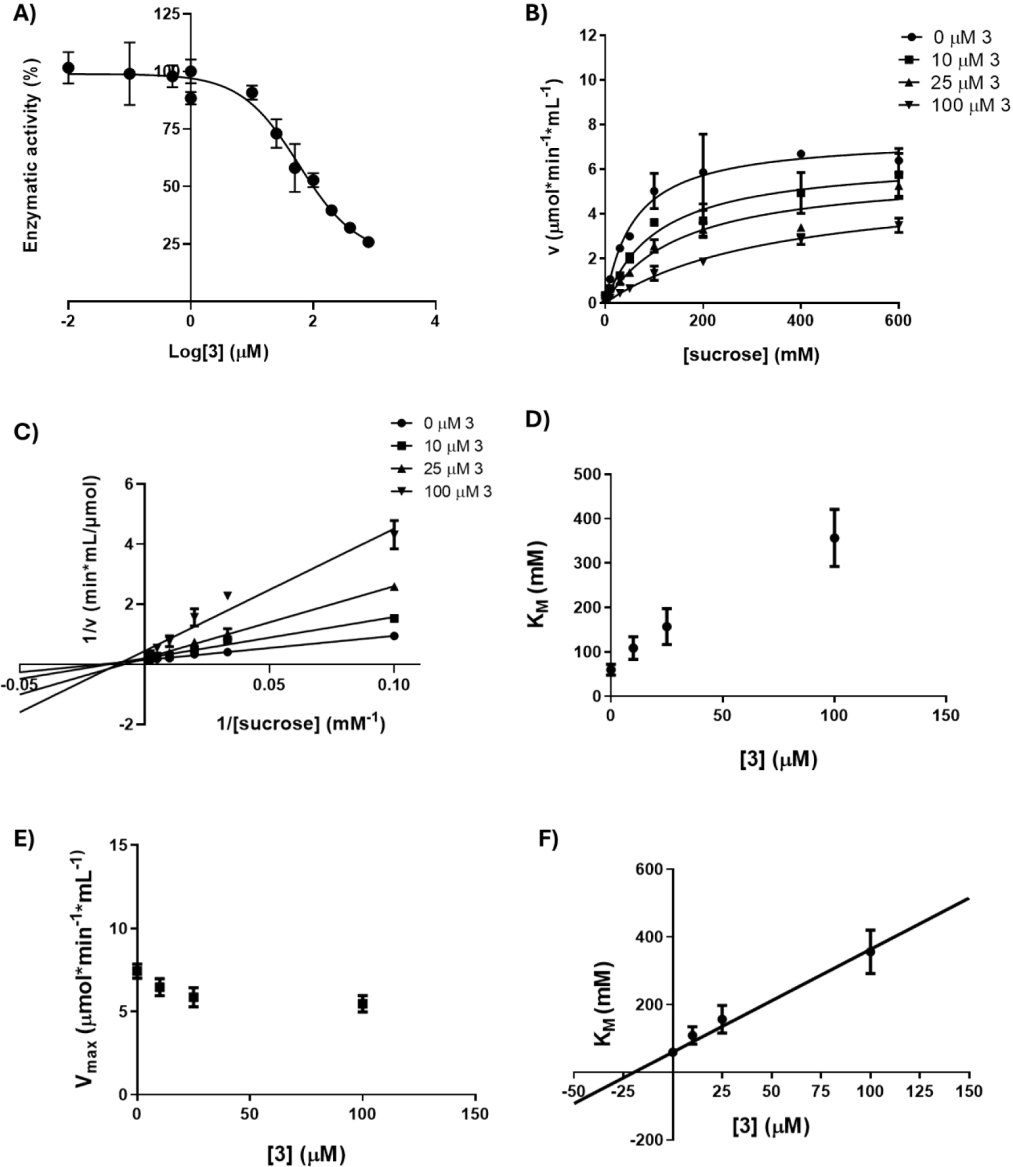
A) IC_50_ of TPIS (**3**) on sucrose hydrolysis of Lscγ. B) Michaelis–Menten plot at three different concentrations of TPIS (**3**) for Lscγ (0, 10, 25, and 100 μM). C) Lineweaver–Burk plot for the detection of kinetic parameters *K*_Mapp_ and *V*_maxapp_ for Lscγ. D) *K*_Mapp_ values of Lscγ at three different concentrations of TPIS (**3**). E) *V*_maxapp_ values of Lscγ at three different concentrations of TPIS (**3**). F) Secondary plot for *K*_i_ determination. Data represent the mean of two different experiments performed in duplicate.

### Structural Characterization of Lscβ

To provide a structural rationale for the multivalent effect observed in the enzyme inhibition by TPIS (**3**), we first determined the crystallographic structure of recombinant Lscβ in its apo form.^16^ The Lscβ structure closely resembles that of other levansucrases, with the five-blade β-propeller topology typical of the glycoside hydrolase family GH68 (Figure 6A).^3,8–11^ Among the levansucrases of known structure, the enzyme from E. tasmaniensis (EtLsc) has the highest sequence identity (78.3%) with Lscβ, as it results also from the belonging of EtLsc and P. syringae levansucrases to the same subfamily 2 of the GH68 family (https://www.cazy.org/GH68_characterized.html). This high sequence identity is reflected in the high structural homology (rmsd = 0.82 Å, considering the Cα atoms of the two proteins), as shown in Figure 6B. The main differences between the two enzyme structures are in the loop comprising residues 90–105 (Lscβ numbering), which has a similar conformation in the two proteins but is slightly rotated in Lscβ with respect to EtLsc. Moreover, the first 16 amino acids of Lscβ do not have a counterpart in the EtLsc sequence. Nevertheless, this N-terminal region of Lscβ was not introduced in the model due to a lack of electron density. The active site is structurally conserved, with the catalytically essential residue Asp62, the nucleophile Asp219, and the acid/base catalyst Glu303^8^. Furthermore, the residues that compose the substrate-binding site are fully conserved with respect to EtLsc, and their side chains have similar conformations and orientations. Whereas, both enzymes present differences in this region compared to SacB: Ser164/Ala148, Arg360/His321, and Glu340/Gln301 (SacB/Lscβ numbering). These differences probably reflect the belonging of SacB to GH68 subfamily 1, instead of the subfamily 2. Notably, there are two monomers in the asymmetric unit, suggesting a dimeric state of the enzyme in agreement with dynamic light scattering and gel filtration data reported by Luti et al. 2021,^16^ and confirming the pivotal role played by the multimeric nature of the enzyme in accepting multivalent ligands.^31,32,49–51^

**Figure 6:**
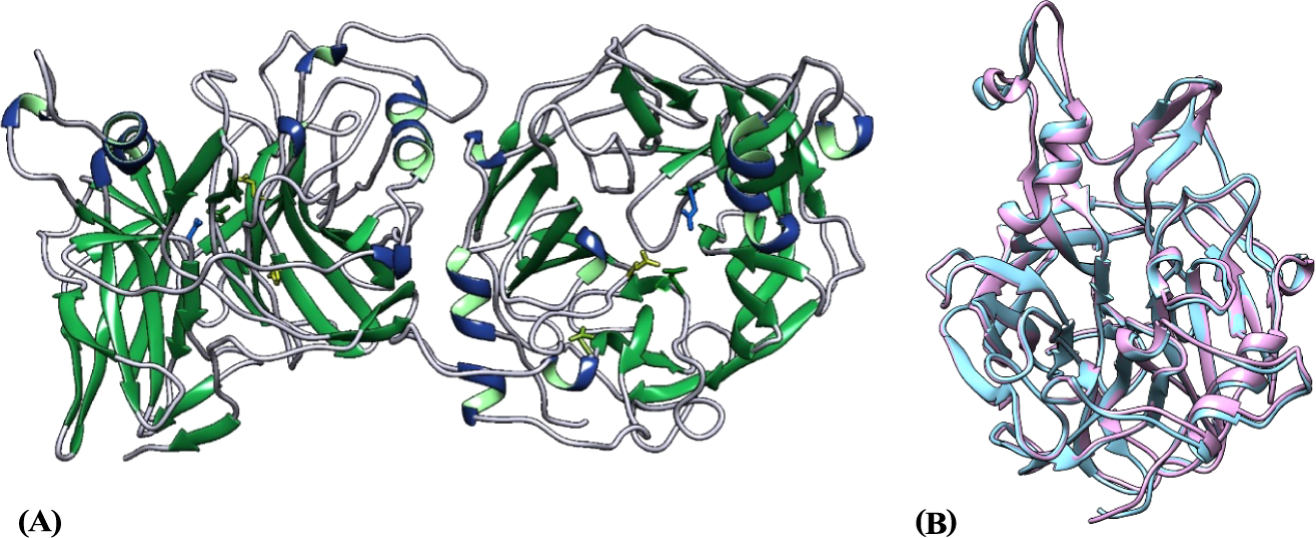
(A) Ribbon diagram of the crystallographic structure of dimeric Lscβ. The catalytic residues are also shown as a stick model. (B) Superposition of the structure of one subunit of apo Lscβ (PDB ID 8QJ5, colored in pink) with the structure of the levansucrase from E. tasmaniensis (PDB ID 7OSO, colored in light blue).

### Structural Characterization of Lscβ in Complex with Inhibitor TPIS (**3**)

Although X-ray structures of glycosidases in complex with multivalent iminosugars are scarce in the literature^52–54^ probably due to ligand flexibility, we succeeded in obtaining the crystal structure of Lscβ complexed with TPIS (**3**) (Figure 7A). The binding mode of the pyrrolidine iminosugar in TPIS (**3**) reflected that previously described for the fructose moiety in the structure of levansucrase mutants of B. subtilis and Brenneria sp. in complex with sucrose (Figure 7B).^1,3^ This confirms that the pyrrolidine iminosugar moiety in TPIS (**3**) mimics well the fructose moiety in sucrose.

**Figure 7:**
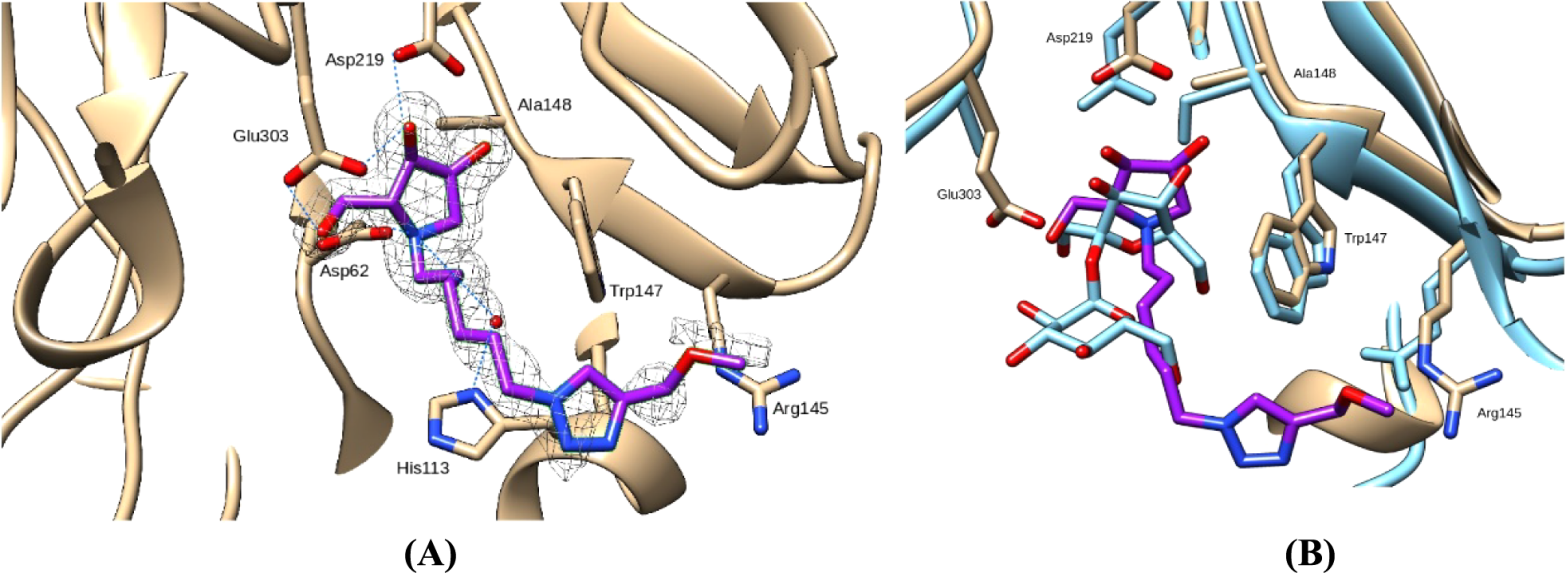
(A) Active site of Lscβ in complex with TPIS (**3**). The *F*_o_–*F*_c_ map is also shown contoured at 2.5 σ. Dotted lines represent hydrogen bonds. (B) Active site superposition of the Lscβ/TPIS (**3**) complex with the sucrose complex of levansucrase from B. subtilis (PDB ID 1PT2).

In agreement with the competitive mechanism highlighted by kinetic studies, a first binding site was observed at the active site. The hydroxyl groups of one iminosugar unit of TPIS (**3**) establish hydrogen bonds with the peptide nitrogen of Ala148 and the side chains of catalytic residues Asp219 and Glu303. The endocyclic nitrogen of the same iminosugar unit forms a hydrogen bond with a water molecule that in turn is bound to His113, and a salt bridge with Asp62, considering the nitrogen protonated. The alkyl chain attached to the above-mentioned iminosugar endocyclic nitrogen forms Van der Waals contacts with Trp147, and the ethereal oxygen makes hydrogen bonds with Arg145 and Thr168. The electron density was absent for the remaining part of the tetravalent inhibitor, suggesting a certain degree of disorder relative to the other three iminosugar-ending tails of TPIS (**3**), which are likely to occupy multiple conformations. A second molecule of TPIS (**3**) binds an alternative secondary binding site located at the interface between two different dimers inside the crystal lattice, approximately 30 Å far from the active site (Figure S10). Also in this site, only a single chain of the tetravalent inhibitor was visible in the electron density maps. The crystal structure of the enzyme/inhibitor complex provided useful information on the mechanism responsible for the inhibitory activity of TPIS (**3**). In fact, the structural disorder of three out of four iminosugar-ending tails of the inhibitor not bound to the active site allows us to exclude strong additional interactions with noncatalytic subsites that could reinforce the binding, providing an inhibitor potency increase, as observed, for example, in the complex of a bacterial fucosidase with a divalent iminosugar.^52^ On the other hand, our complex structure does not support aggregative processes, as confirmed also by dynamic light scattering experiments (Figure 8), leading us to exclude the formation of cross-linked networks promoted by TPIS (**3**), as reported for the multimeric Jack bean α-mannosidase complexed with a multivalent inhibitor,^31^ nor the formation of clusters.^53^ The two active sites, which are deeper compared to those of α-mannosidases and do not face one another in the dimer, are 55 Å apart, a much greater distance than that between two iminosugar-ending tails of TPIS (**3**) (roughly 25 Å). Therefore, simultaneous occupation of the two active sites of a dimer by two branches of a single molecule of TPIS (**3**) is unlikely. In summary, the analysis of the crystal structure of the enzyme/inhibitor complex confirms TPIS (**3**) as a competitive inhibitor of levansucrase(s), as emerged from kinetic data, and indicates that among the mechanisms potentially accounting for the positive multivalent effect observed, statistical rebinding seems the most plausible, although other effects cannot be ruled out.

**Figure 8:**
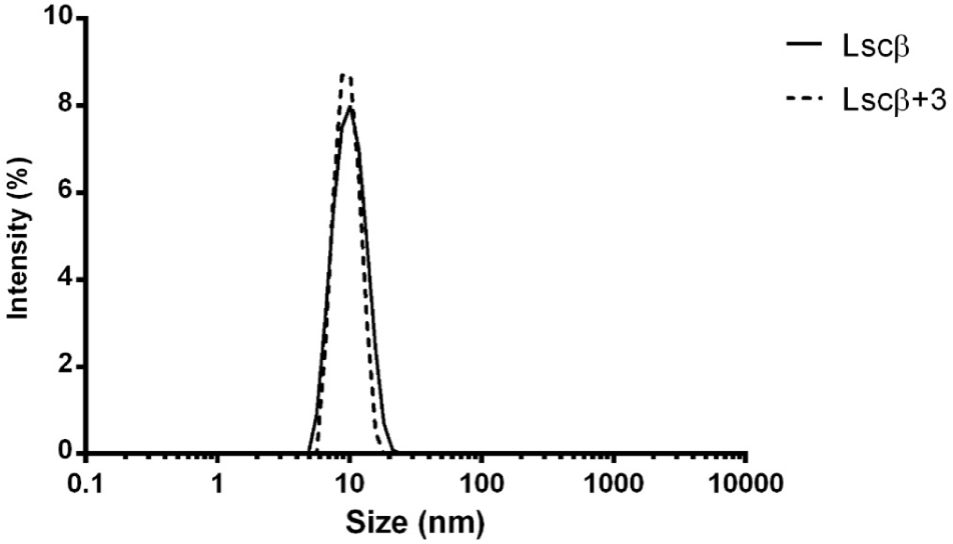
Particle size distributions of Lscβ alone or with 100 μM TPIS (**3**). DLS experiment was performed at pH 7.0 in 50 mM Bis/Tris buffer. Data are the mean of three different accumulations.

### Evaluation of the Inhibitory Activity of TPIS (**3**) on Levan Polymerization

In a previous work, we demonstrated that Lscβ and Lscγ show the maximum polymerization activity at pH 5.0 and that the polymerization rate of Lscγ is higher than Lscβ.^16^ Therefore, we chose Lscγ to evaluate levan synthesis inhibition by turbidity assay.^16,55^ Figure 9A confirms that TPIS (**3**) at a 100 μM concentration inhibits levan production by Lscγ with 46 ± 3% of residual activity. We used the same turbidity assay to estimate the IC_50_ adding different TPIS (**3**) concentrations, ranging from 0.781 to 200 μM. The results obtained are reported in Figure 9B. In Figure 9C, we report the percentage of enzyme residual activity calculated at 60 min of incubation, which is the point of maximum absorbance. This procedure allowed us to estimate the IC_50_ for the polymerization activity of Lscγ at pH 5.0 (37 μM).

**Figure 9:**
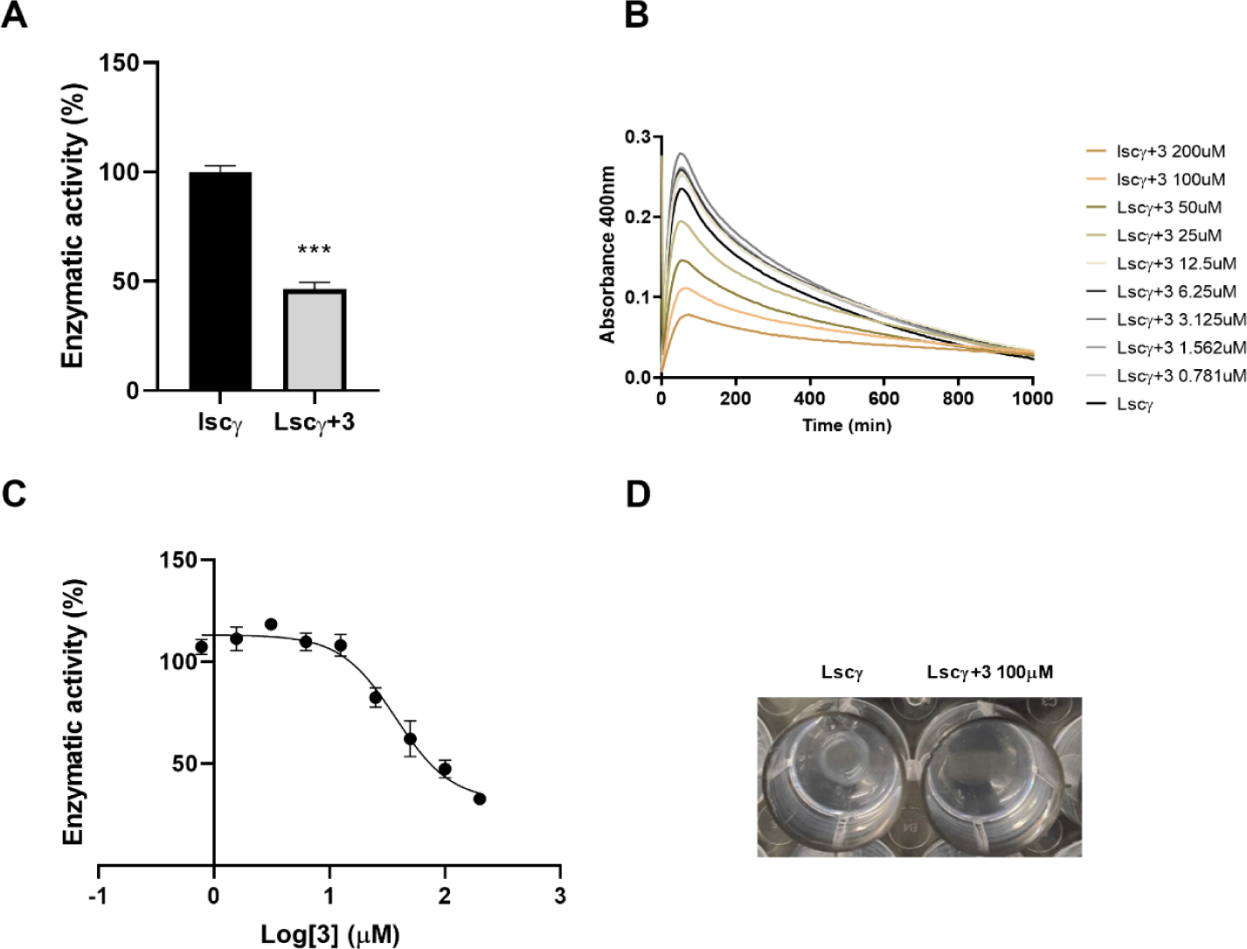
Activity of TPIS (**3**) on levan synthesis by Lscγ. (A) Residual polymerization activity of Lscγ at 100 μM iminosugar, pH 5.0. (B) Time course of levan polymerization by Lscγ alone or with different concentrations of TPIS (**3**). (C) IC_50_ of TPIS (**3**) on the levan synthesis of Lscγ at pH 5.0. (D) levan synthesis by a 10 μL drop of 1 μg/μL Lscγ applied on an agar sucrose plate alone or with 100 μM of TPIS (**3**). Data are the mean of three experiments. *t* test (*** *p* ≤ 0.001).

Finally, to have a macroscopic evaluation of levan synthesis inhibition, a 10 μL drop of 1 μg/μL Lscγ was applied on an agar sucrose plate alone or with 100 μM of TPIS (**3**). Results clearly show that the addition of TPIS (**3**) to the sucrose plate limits levan production, as demonstrated by the absence of the polymer disc (Figure 9D).

### Spectrum of Inhibition on Different P. Syringae Lysates

Finally, to explore the spectrum of inhibition of TPIS (**3**), we checked sucrose hydrolysis and levan polymerization inhibition on cell lysates derived from P. syringae pv actinidiae biovar 5 (CFBP8414) and P. syringae pv phaseolicola (CFBP1390), both of which are levan-positive bacteria phylogenetically close to Psa3.^16,56^ Figure 10A indicates that the addition of 100 μM TPIS (**3**) reduces the sucrose hydrolysis rate in a similar fashion in all cell lysates: KL103, 54 ± 7% of residual activity; CFBP8414, 44 ± 11% of residual activity; CFBP1390, 55 ± 3% of residual activity, confirming TPIS (**3**)’s effectiveness against a wide range of strains. Moreover, on the same lysates, TPIS (**3**) is able to limit levan synthesis, as demonstrated by the macroscopic assay of levan production reported in Figure 10B. Overall, these results demonstrate that: (i) TPIS (**3**) is able to affect both the hydrolysis and polymerization reactions of levansucrases; (ii) it is able to reduce Lscβ and Lscγ activity not only on the purified recombinant enzymes but also using Psa3 cell lysates; and (iii) it has efficacy against the levansucrases produced by the three tested strains of P. syringae, a cosmopolitan species for which more than 13 phylogroups and 64 pathovars are currently recognized.^57^

**Figure 10:**
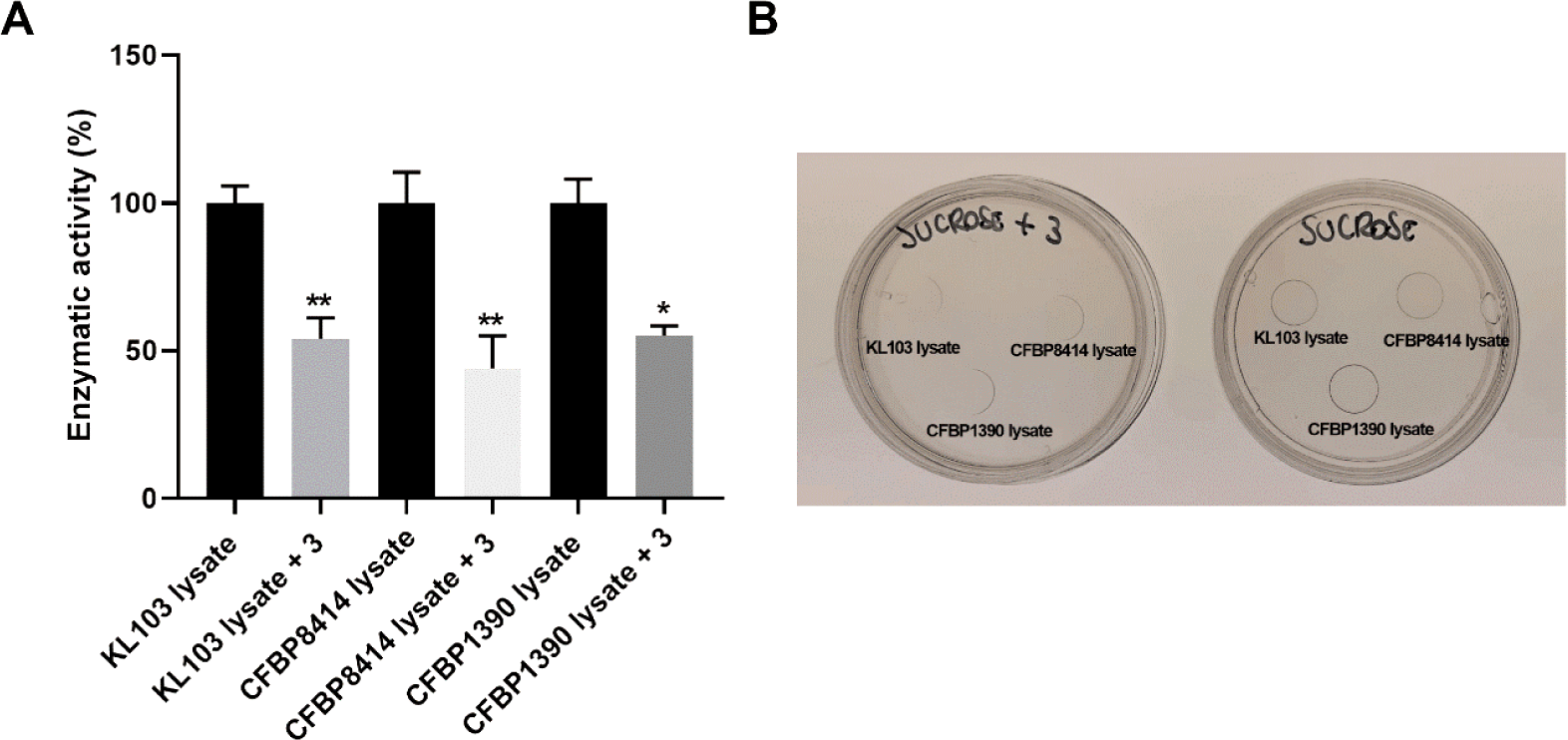
(A) Residual sucrose hydrolysis activity of whole cell lysates alone or with 100 μM TPIS (**3**). (B) Levan synthesis by a 10 μL drop of P. syringae KL103, CFBP8414 and CFBP1390 whole cell lysates applied on agar sucrose plate alone or with 100 μM TPIS (**3**). Data are the mean of 3 experiments. One-way ANOVA (* *p* ≤ 0.05; ** *p* ≤ 0.01).

In conclusion, levansucrases are bacterial enzymes that can use sucrose as a substrate for catalyzing the hydrolysis of sucrose into glucose and fructose, the latter acting as an acceptor during levan biosynthesis. The tetravalent pyrrolidine iminosugar TPIS (**3**) herein identified is the first levansucrase inhibitor described to date. It is able to reversibly inhibit both Lscβ and Lscγ isoforms recombinantly produced starting from the *lsc* genes of P. syringae pathovar actinidiae biovar 3, at micromolar concentrations and with a competitive binding mode. The crystal structure of Lscβ complexed with TPIS (**3**) confirmed the competitive nature of the inhibition and suggested a mechanism for the strong reduction in sucrose hydrolysis by TPIS (**3**), in which one of the four pyrrolidine iminosugar units interacts with the active site of the enzyme and the other three branches favor a statistical rebinding effect. Interestingly, TPIS (**3**) is able to reduce Lscβ and Lscγ activity not only on the purified enzymes but also using Psa3 cell lysates, demonstrating levansucrase inhibition activity also in a complex protein environment. Finally, TPIS (**3**) shows activity on both hydrolysis and polymerization reactions on different levansucrase isoforms derived from 2 pathovars of P. syringae (pv actinidiae and phaseolicola) and from 2 biovars of P. syringae pv actinidiae (biovars 3 and 5). Further investigations are compelling to elucidate the role of TPIS (**3**) in altering the host–pathogen relations on Psa3. Since levan is suggested to play an important role in bacterial fitness and in plant–pathogen interaction,^14^ the identification of a strong inhibitor of enzymes responsible for levan synthesis could be useful in understanding the function of this polyfructan polymer in regulating bacterial growth in different conditions as well as in the induction of pathogenesis in plants. Moreover, the knowledge at the molecular level of the interaction mechanism between TPIS (**3**) and levansucrase(s) paves the way to the development of new compounds capable of inhibiting the synthesis of levan and consequently inducing alterations in the plant–pathogen recognition mechanism in bacteria of the P. syringae species complex.^57^

## Supplementary Material



## Data Availability

The raw data supporting the conclusions of this manuscript will be made available by the authors to any qualified researcher.

## Notes

Sequence alignment of P. syringae (pv actinidiae) lenvansucrases Lscβ and Lscγ, details of compound **1**–**4** used in this study, statistics of the crystallographic data collections and Figure S10PDF)

## Abbreviations:

DAB-1: 1,4-dideoxy-1,4-imino-d-arabinitol^29^
Psa3: Pseudomonas syringae pv actinidiae biovar 3
GH68: glycosidase 68 family
TPIS: tetravalent pyrrolidine iminosugar
PIS: pyrrolidine iminosugar
