# Supplementary material for: Molecular Basis of Pseudomonas syringae pv actinidiae Levansucrase Inhibition by a Multivalent Iminosugar
Source: J Agric Food Chem. 2025 May 11;73(25):15981–92. doi: 10.1021/acs.jafc.5c01947 (PMC12203577; doi:10.1021/acs.jafc.5c01947)
Supplement: Supplementary file 1 [file jf5c01947_si_001.pdf]

Supporting Information

Molecular basis of *Pseudomonas syringae* pv. *actinidiae* levansucrase inhibition by a multivalent iminosugar

Costanza Cicchi,[a] Luigia Pazzagli,[a] Paolo Paoli, [a] Sara Campigli,[b] Guido Marchi,[b] Francesca Cardona,[c] Francesca Clemente,[c] Sara Pavone,[c] Marta Ferraroni,*[c] Alberto Canovai,[c] Camilla Matassini*[c] and Simone Luti*[a]

[a] Department of Experimental and Clinical Biomedical Sciences, University of Florence, Viale Morgagni n. 50, 50134 Florence, Italy

[b] Department of Agriculture, Food, Environment and Forestry, University of Florence, Piazzale delle Cascine n. 28, 50144, Florence, Italy

[c] Department of Chemistry 'Ugo Schiff' (DICUS), University of Florence, via della Lastruccia n. 3-13, Sesto Fiorentino (FI), 50019, Italy

Table of Content

- Sequence alignment of *P.syringae* (pv. *actinidiae*) lenvansucrases Lscβ and Lscγ S3
- Characterization of compounds **1-4** S4
- X-ray analysis (Table S1) S9
- Figure S10 S10
- References S11

Sequence alignment of *P. syringae* (pv. *actinidiae*) lenvansucrases Lscβ and Lscγ

```
Lscγ        -MIAGRRHFDCRPLHLAGNINYEPTVWSRADALKVNENDPTTTQPLVSADFPVMSDTVFI  59
Lscβ        MSTSSSALSQLKNSPLAGNINYEPTVWSRADALKVNENDPTTTQPLVSADFPVMSDTVFI  60
               :.    : :   *********************************************

Lscγ        WDTMPLRELDGTVVSVNGWSVILTLTADRHPNDPQYLDANGRYDIKRDWEDRHGRARMCY  119
Lscβ        WDTMPLRELDGTVVSVNGWSVILTLTADRHPDDPQYLDANGRYDIKRDWEDRHGRARMCY  120
            *******************************:****************************

Lscγ        WYSRTGKDWIFGGRVMAEGVSPTTREWAGTPILLNDKGDIDLYYTCVTPGAAIAKVRGRI  179
Lscβ        WYSRTGKDWIFGGRVMAEGVSPTTREWAGTPILLNDKGDIDLYYTCVTPGAAIAKVRGRI  180
            ************************************************************

Lscγ        VTSDQGVELEDFTLVKKLFEANGTYYQTEAQNSSWNFRDPSPFIDPNDGKLYMVFEGNVA  239
Lscβ        VTSDQGVELKDFTQVKKLFEADGTYYQTEAQNSSWNFRDPSPFIDPNDGKLYMVFEGNVA  240
            *********:*** *******:**************************************

Lscγ        GERGSHTVGAAELGPVPPGHEDVGGARFQVGCIGLAVAKDLSGEEWEILPPLVTAVGVND  299
Lscβ        GERGSHTVGAAELGPVPPGHEDVGGARFQVGCIGLAVAKDLSGEEWEILPPLVTAVGVND  300
            ************************************************************

Lscγ        QTERPHYVFQDGKYYLFTISHKFTYAEGLTGPDGVYGFVGEHLFGPYRPMNASGLVLGNP  359
Lscβ        QTERPHYIFQDGKYYLFTISHKFTYAEGLEGPDGVYGFVGEHLFGPYRPMNASGLVLGNP  360
            *******:********************* ******************************

Lscγ        PEQPFQTYSHCVMPNGLVTSFIDSVPTEGEDYRIGGTEAPTVRILLKGDRSFVQEEYDYG  419
Lscβ        PEQPFQTYSHCVMPNGLVTSFIDSVPTDGEDYRIGGTEAPTVRIVLKGDRSFVQEEYDYG  420
            ***************************:****************:***************

Lscγ        YIPAMKDVTLS   430
Lscβ        YIPAMKDVQLS   431
            ******** **
```

Figure S1. The image presented here is adapted from a Clustal Omega alignment of the two levansucrases sequences. The differences in amino acid sequences are highlighted in yellow.

Characterization of compounds 1-4

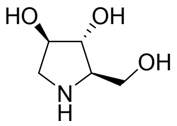

DAB-1 **(1)**

Elemental analysis calcd (%) $C_5H_{11}NO_3$ (133.12): C 45.10, H 8.33, N 10.52; found: C 44.82, H 8.11, N 10.23.

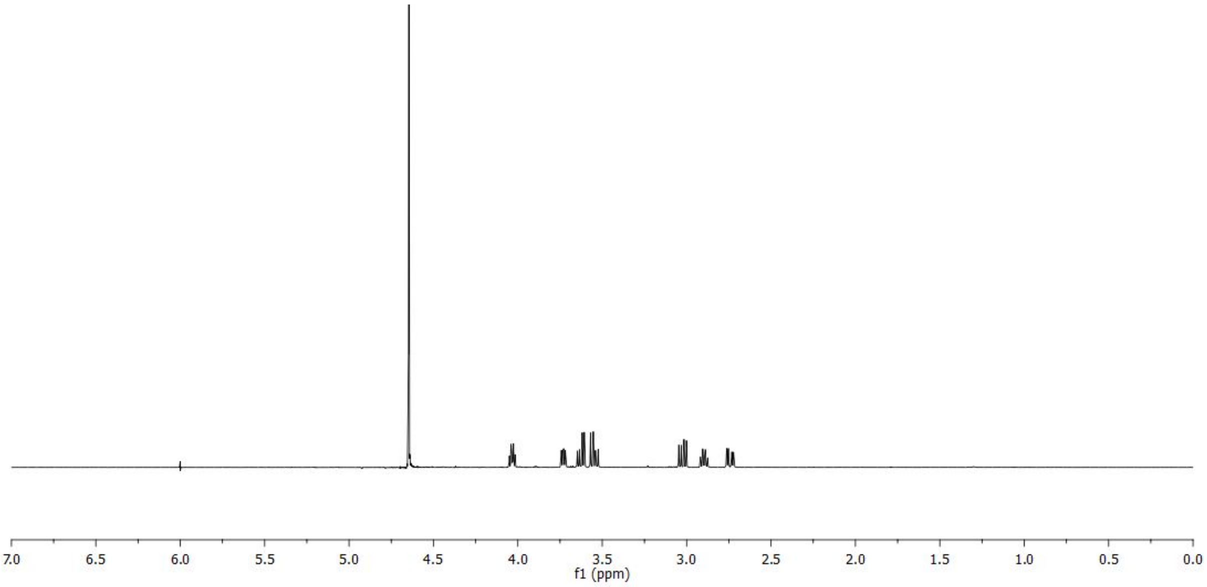

Figure S2. ^{1}H-NMR spectrum of compounds **1** (400 MHz, D_2O)

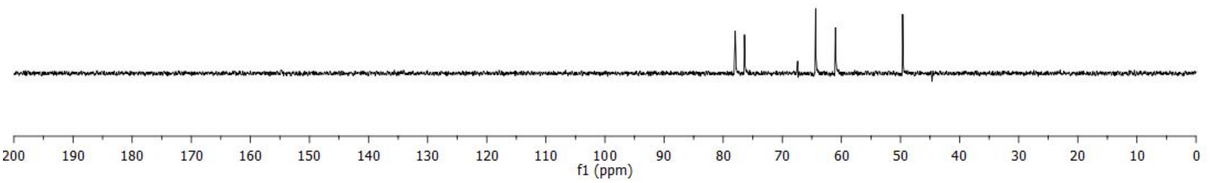

Figure S3. ^{13}C-NMR spectrum of compounds **1** (50 MHz, D_2O)

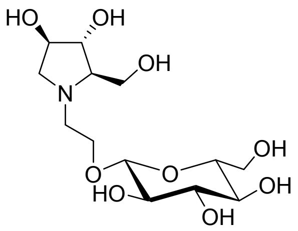

2

Elemental analysis calcd (%) for $C_{13}H_{25}NO_9$ (339.34): C 46.01, H 7.43, N 4.13; found: C 46.67, H 7.45, N 3.20.

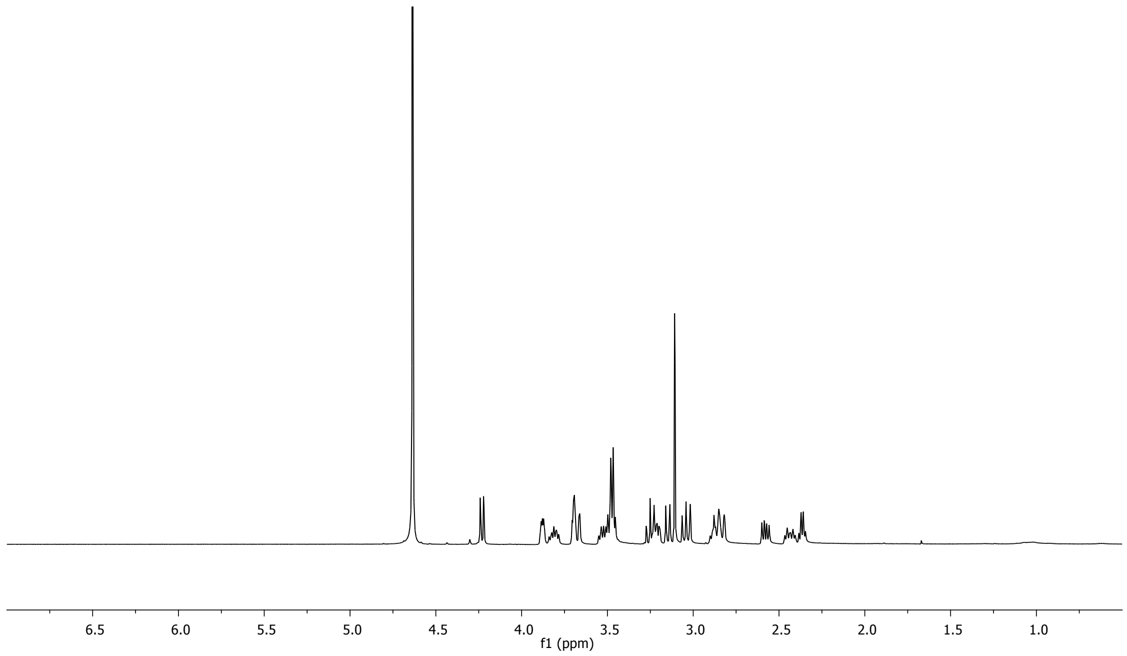

Figure S4. ^{1}H-NMR spectrum of compounds **2** (400 MHz, D_2O)

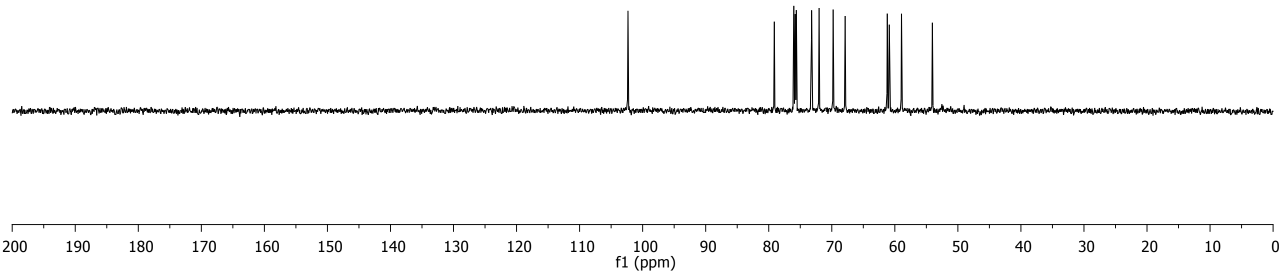

Figure S5. ^{13}C-NMR spectrum of compounds **2** (100 MHz, D_2O)

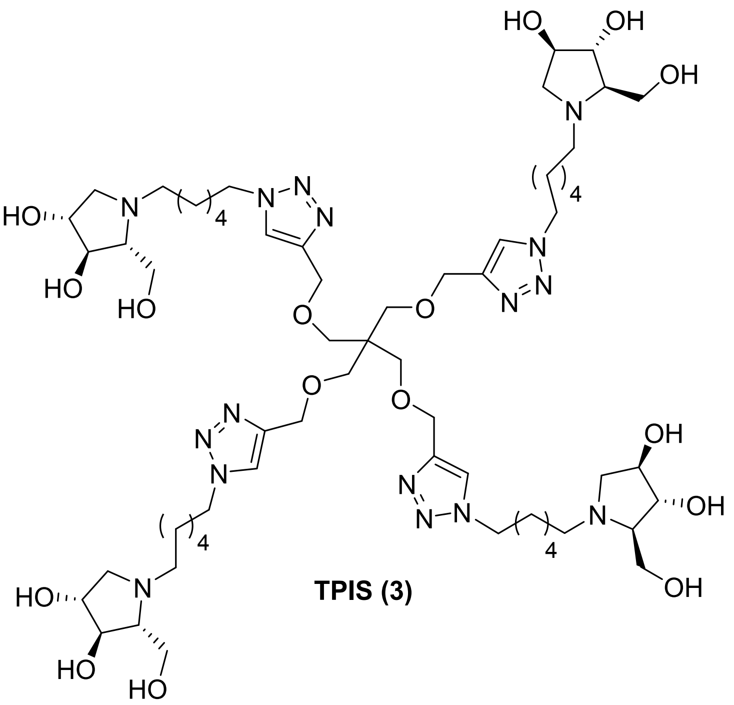

TPIS (3)

Elemental analysis calcd (%) for $C_{61}H_{108}N_{16}O_{16}$ (1321.61): C 55.44, H 8.24, N 16.96; found: C 55.75, H 8.12, N 16.63.

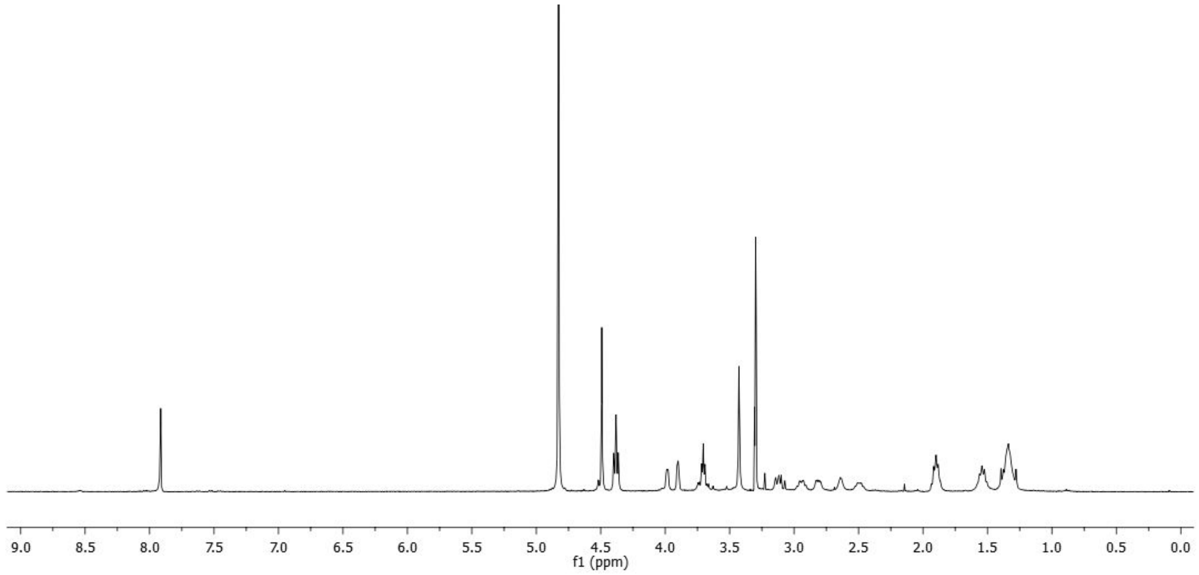

Figure S6. ^{1}H-NMR spectrum of compounds TPIS (**3**) (400 MHz, CD_3OD)

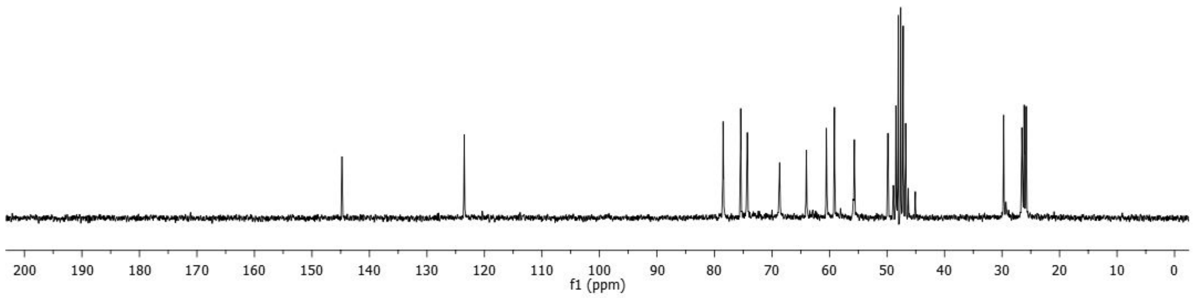

Figure S7. ^{13}C-NMR spectrum of compounds TPIS (**3**) (50 MHz, CD_3OD)

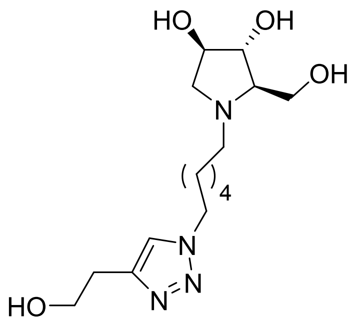

PIS (4)

Elemental analysis calcd (%) for $C_{15}H_{28}N_4O_4$ (328.41): C 54.86, H 8.59, N 17.06; found: C 54.79, H 8.57, N 17.09.

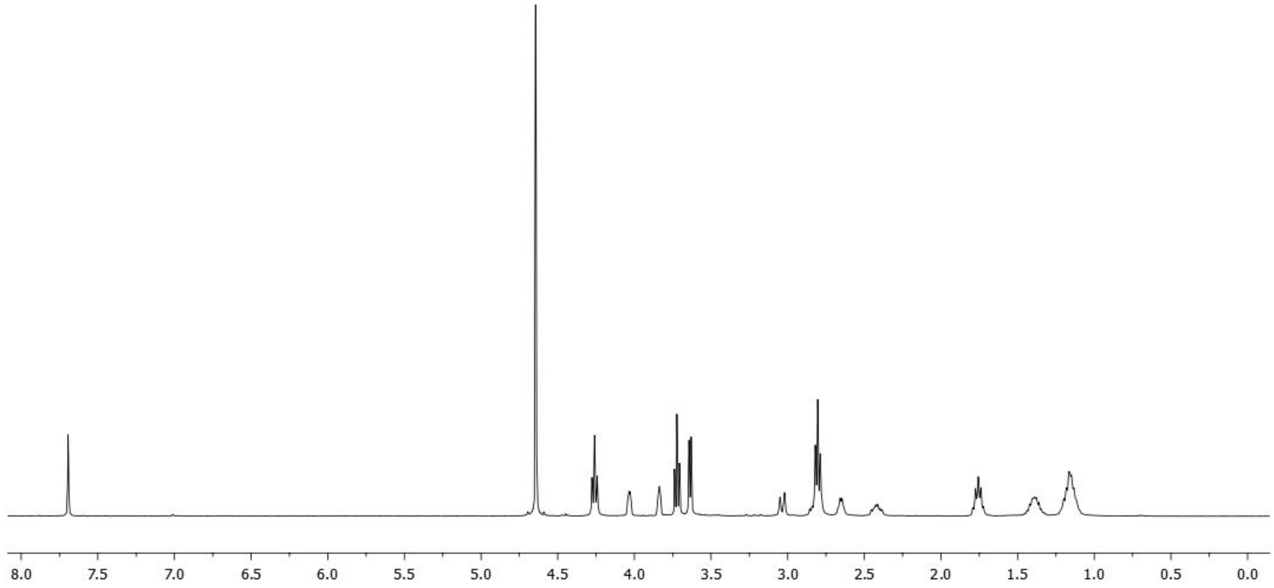

Figure S8. ^{1}H-NMR spectrum of compound PIS (**4**) (400 MHz, D_2O)

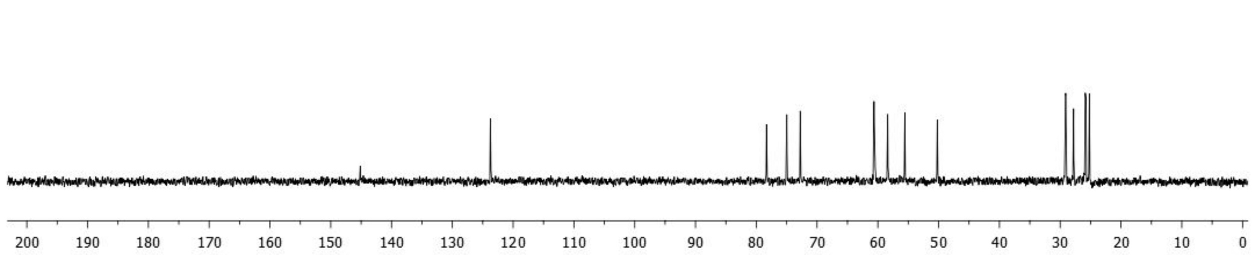

Figure S9. ^{13}C-NMR spectrum of compound PIS (**4**) (50 MHz, D_2O)

X-ray analysis

Table S1. Summary of Data Collection and Refinement Statistics. Values in parentheses refer to the highest resolution shell.

	**Lscb native**	**Lscb + TPIS (3)**
PDB ID	8QJ5	8QKW
Wavelength (Å)	1.0000	1.0000
Space Group	$P2_1$	*C*2
Unit cell (a, b, c, β) (Å, °)	57.61, 100.63, 77.93, 96.06	120.09, 63.99, 67.99, 103.5
Limiting resolution (Å)	29.31 – 1.63 (1.67 – 1.63)	39.44 – 1.65 (1.69 - 1.65)
Unique reflections	702500 (35800)	59642 (4378)
Rmerge (%)	7.7 (104.3)	8.2 (97.0)
Rmeas (%)	8.3 (113.4)	9.0 (105.2)
Redundancy	6.4 (6.5)	6.5 (6.7)
Completeness overall (%)	99.8 (100)	98.6 (97.7)
<I/σ(I)>	14.9 (2.1)	12.9 (2.2)
CC (1/2) %	99.9 (77.1)	99.8 (74.1)
**Refinement statistics**		
Resolution range (Å)	29.31 – 1.63	39.44 – 1.65
Rfactor (%)	16.11	15.53
Rfree(%)	19.03	18.69
r.m.s.d. bonds(Å)	0.0112	0.0123
r.m.s.d. angles (°)	1.812	1.933
**Ramachandran statistics** (%) *		
Most favored	96.3	96.3
additionally allowed	3.4	3.2
outlier regions	0.2	0.5
**Average B factor (Å^2)**		
All atoms	24.08	25.15
inhibitors	-	50.8
solvent	34.76	33.43

* Calculated with the program Rampage[1] from the CCP4 package.

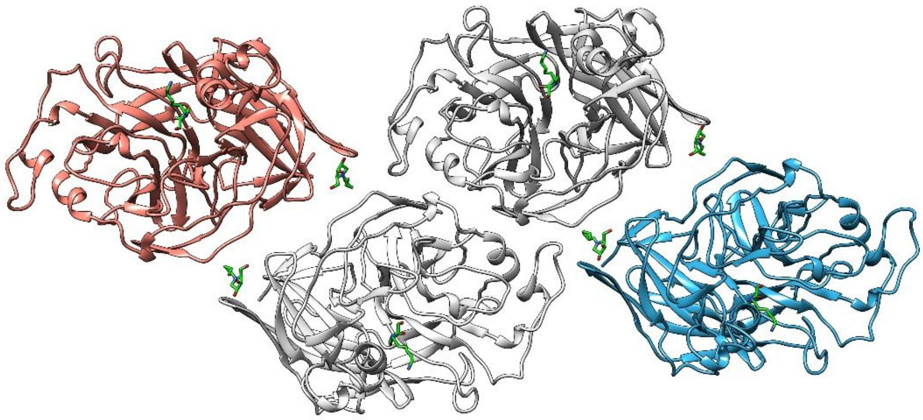

Figure S10. Ribbon diagram of the dimeric Lscβ/TPIS (**3**) complex (colored in gray) showing also two symmetry related monomers and the position of the inhibitor bound to the active sites and to the secondary binding sites at the interface between two dimers.

References

1. Lovell, S.C., Davis, I.W., Arendall, W.B., de Bakker, P.I.W., Word, J.M., Prisant, M.G., Richardson, J.S., and Richardson, D.C. (2003). Structure validation by Cα geometry: ϕ,ψ and Cβ deviation. Proteins Struct. Funct. Bioinforma. *50*, 437–450. 10.1002/prot.10286.
